# Responsibility Revisited

**DOI:** 10.1007/s12376-012-0077-9

**Published:** 2012-05-17

**Authors:** Silke Schicktanz, Aviad Raz

**Affiliations:** 1 Department of Medical Ethics and History of Medicine, University Medical Center Goettingen, Humboldtallee 36, Goettingen, Germany; 2 Department of Sociology and Anthropology, Ben Gurion University of the Negev, P.O. 653, Beersheba, Israel

Recent developments in medicine open up new possibilities for planning and shaping life. At the same time, this scope of new options and interventions also involves new forms and spheres of responsibilities. Elderly persons can be viewed as having a responsibility toward their families and partners to plan, via advance health care directives, the final stages of their life; individuals can be seen as responsible for late onset diseases when ignoring public incitements for a healthy life style; and medical professionals can be regarded as responsible for “wrongful” lives.

These new forms of responsibility concern medical professionals, patients, families, and even society in general. The emerging idea of “responsibilisation” by the new politics of “life itself”—as Rose (2006) termed it—warrants more attention and reflection. However, in bioethics, the term is notoriously unclear. This thematic issue of *Medicine Studies* tries therefore to explore the multiple meanings of responsibility in the context of biomedical practices in an interdisciplinary perspective.

The five contributions to this special themed issue of Medicine Studies draw on a range of examples and disciplines to explore responsibility as a concept that reaches beyond discussions of autonomy to everyday aspects of relational ethics. *Silke Schicktanz and Mark Schweda* provide a general point of departure by developing a philosophical–analytical formulation of responsibility in medicine, aiming for an in-depth understanding and critique of moral claims on a meta-ethical level without presuming one particular normative approach. *Carmel Shalev* focuses on the social responsibility of individuals in the context of assisted reproduction technologies, reminding us how this seemingly liberating project of rights should also involve empathetic self-reflection regarding the role of third-party women who participate in this transaction. *Carlo Petrini and Michele Farisco* highlight the complex legal definitions of professional–clinical responsibility, and the limits of legal frameworks as professional–ethical questions are going beyond them. *Maria Hedlund* shows us the political aspects of responsibility in the context of epigenetics. For her, new insights into the environmental impact on our genetic constitution are good reasons to change the course from blaming citizens to social activities of protection. Finally, *Signe Mezinska, Ilze Mileiko, and Aivita Putnina* bring together several perspectives of responsibility in their anthro-ethical study of gamete donation in Latvia.

An intriguing duality of responsibility underlies and interconnects these various papers. The papers show us that, importantly, responsibility in the biomedical context is relational and contextual, involving various facets such as self-responsibility, responsibility for kin, as well as the responsibility for society and of society. In addition to the different underlying subject–object relationships, we can also construe forward and backward forms of responsibility. All the papers demonstrate that “responsibility” in the biomedical context goes beyond legal responsibility and the question who is to blame for; it also includes issues of emotional relations, feelings, social rights, and political duties, dealing with accountability, vulnerability, and insecurity.

Analyzing responsibility therefore hinges on an empirical understating of why certain people consider certain issues, under certain circumstances, as involving responsibility. In addition, however, the concept of responsibility is not all personal and idiosyncratic; it is framed by broader sociocultural and ethical narratives. Our concepts of responsibility are embedded within different cultural grammars, interconnecting formality and relationality. In English/Latin etymology, responsibility denotes an individual emphasis on self-determination: a responsible person is someone who is “answerable,” that is, who *responds* to accusations raised in front of a court or in parliament, whereas in Hebrew etymology, responsibility reflects *relational support*, as in standing behind someone. Such dialectical focus on the cultural grammars behind individual and interpersonal concepts of responsibility can provide an intriguing interface for bridging some of the gaps between experts’ formal ethics of principles and our lay moralities, between ground and surface rules of moral action, and between theoretical and empirical bioethical analysis.

We see this special issue as providing a drive for and an illustration of the dual understanding of the social narratives and cultural grammars of responsibility that embody personal accounts of responsibilities within action-driven storytelling of actual people in concrete situations. Such hermeneutic and constructivist view has already propelled, in recent years, a new agenda for the so-called “empirical” ethics in medicine studies. This thrust is currently maturing by locating cross-cultural embeddings of moral contentions of responsibility, such as the duty to know and the right not to know one’s genetic dispositions, or the sanctity of life versus self-determination in the context of end-of-life medical care. As medicine increasingly accompanies our lives, from beginning to end, current and future research seeks to understand how people understand responsibilities in the context of cultural factors such as religion, history, utopian, and dystopian views of biomedicine, outlooks on the body and on health/illness, consumerism, and individualism/collectivism (Raz and Schicktanz 2013). We hope this issue will propel further studies of responsibility that interconnect cultures, forms of sociality, subjectivities, and identities, all integrated by a bioethics that is sensitive to its socioempirical moment. This special themed issue emanated from our continuing comparative studies of health and responsibility in Germany and Israel, and we are grateful to Prof. Norbert Paul for encouraging us to pursue this project.
